# C-reactive protein velocity and inflammatory burden index: new systemic inflammatory biomarkers and their predictive value for the latent period in preterm premature rupture of membrane pregnancies

**DOI:** 10.1007/s00404-025-08089-1

**Published:** 2025-06-28

**Authors:** Dilara Duygulu Bulan, Bengu Mutlu Sutcuoglu, Gulsan Karabay, Zeynep Seyhanli, Nazan Vanli Tonyali, Halis Dogukan Ozkan, Sevki Celen

**Affiliations:** 1 Department of Obstetrics and Gynecology, Division of Perinatology, Ankara Etlik City Hospital, 06170 Ankara, Turkey; 2 Department of Obstetrics and Gynecology, Ankara Atatürk Sanatorium Training and Research Hospital, Ankara, Turkey; 3 Department of Obstetrics and Gynecology, Ankara Lösante Children and Adult Hospital, Ankara, Turkey

**Keywords:** C-reactive protein velocity, Inflammatory burden index, Preterm premature rupture of membranes, Latent period, Inflammatory biomarkers

## Abstract

**Objective:**

This study aimed to evaluate the prognostic value of C-reactive protein velocity (CRPv) and inflammatory burden index (IBI) as novel systemic inflammatory biomarkers in predicting the latent period in pregnancies complicated by preterm premature rupture of membranes (PPROM). We investigated the role of these markers in estimating the timing of delivery and their association with perinatal and neonatal outcomes.

**Methods:**

This retrospective cohort study included 337 singleton pregnancies diagnosed with PPROM at Ankara Etlik City Hospital between January 1, 2024, and January 1, 2025. Patients were divided into two groups based on the latency period. Maternal inflammatory markers, CRPv, and IBI, were recorded and analyzed. The relationship between these inflammatory markers and latency period was assessed, and ROC curve analysis was performed to determine optimal cutoff values.

**Results:**

CRPv and IBI values were significantly higher in patients with a latency period of less than 48 h compared to those delivering after 48 h (*p* < 0.001). ROC analysis revealed that CRPv > 0.17 had 70% sensitivity and 67% specificity in predicting delivery within 48 h (AUC = 0.675, *p* < 0.001), while IBI > 35.8 demonstrated predictive accuracy for early delivery (AUC = 0.563, *p* = 0.047).

**Conclusion:**

CRPv and IBI are promising inflammatory biomarkers for predicting the latency period in PPROM pregnancies. Their incorporation into clinical management may improve risk stratification, aiding in timely interventions to optimize maternal and neonatal outcomes. Further prospective studies are warranted to validate these findings and explore their integration into standard obstetric care.

## What does this study adds to the clinical work

**Table:** 

CRPv as a predictor: The study shows that CRPv levels are significantly associated with the probability of delivery within 48 h in PPROM patients. With an AUC of 0.675 and a sensitivity of 70%, CRPv can serve as a reliable biomarker for early identification of patients at high risk of rapid labor, allowing clinicians to make timely decisions regarding monitoring and interventions	
IBI as a management tool: This study reveals that IBI can be a valuable indicator for assessing preterm labor risk and has sensitivity and specificity values that support its potential use in clinical practice. In particular, patients with IBI values >35.8 should be closely monitored for PPROM and prioritized for labor preparation	

## Introduction

Preterm premature rupture of membranes (PPROM) is defined as the spontaneous rupture of fetal membranes before the 37^th^ week of gestation, before the onset of uterine contractions [1]. This condition, which is responsible for one-third of premature births, is a pregnancy complication affecting approximately 3–4% of all pregnancies [2]. PPROM may lead to various neonatal complications including respiratory distress syndrome (RDS), neonatal sepsis and intraventricular hemorrhage (IVH)[3]. The main aim in the follow-up of PPROM patients is to closely monitor the inflammatory process to prevent complications that may arise due to prematurity and to gain the optimal time for betamethasone treatment.

C-reactive protein (CRP) is an important biomarker in protein structure used for early diagnosis of various obstetric complications [4]. Studies have shown an association between high CRP levels and intraamnionic inflammation, but its diagnostic accuracy in predicting complications in PPROM is still controversial [5]. CRP velocity (CRPv) and inflammatory burden index (IBI) are new inflammatory parameters. Among these inflammatory markers, CRPv is obtained by measuring the rate of change of CRP levels over time and this parameter provides more information about the dynamics of the inflammatory process [6]. This parameter has recently gained importance in studies conducted in fields other than obstetrics and gynecology [6, 7]. Although IBI is a biomarker used to evaluate the effects of systemic inflammation and to determine the prognosis of various diseases, our knowledge about its role in predicting obstetric complications is limited.

The interval between rupture of the amniotic sac and delivery has a significant effect on neonatal complications [8]. Factors affecting this time and the relationship of these factors with neonatal outcomes have been the subject of many investigations[9, 10]. However, to our knowledge, there is no study on the role of CRPv and IBI levels in predicting the time of labor and neonatal outcomes in preterm premature rupture of membranes cases. This is the first study to examine the potential of CRPv and IBI in predicting whether labor will occur within 48 h and 7 days in patients with PPROM.

## Materials and methods

### Study design

This study was designed as a retrospective cohort analysis to investigate the prognostic value of CRPv and IBI in pregnant women diagnosed with PPROM who applied to Ankara Etlik City Hospital Perinatology Department between January 1, 2024 and January 1, 2025.

### Selection of case and selection of control

337 singleton pregnant women diagnosed with PPROM were included in the study, and these pregnant women were divided into two groups according to the timing of delivery. 48 h and 7 days were taken as the criterion for the timing of delivery. A power analysis was conducted using G*Power 3.1.9.7 software to assess the statistical power of the study. In the analysis, the effect size was set at moderate (*d* = 0.5), the significance level at 5% (*α* = 0.05), and the test power at 95% (1 − *β* = 0.95). Based on these parameters, the study achieved sufficient statistical power. PPROM was diagnosed by observing active amniotic fluid flow during sterile speculum examination. In cases where the diagnosis was uncertain, placental α-microglobulin test (PAMG-1) (AmniSure® ROM) was performed to confirm the diagnosis [11]. Only patients in the 24–34th gestational week who completed all antenatal examinations and gave birth in our hospital were included in the study. Patients diagnosed with fetal anomalies, maternal chronic disease, maternal medication use and women with multiple pregnancies were excluded. In addition, clinical findings such as maternal fever (≥ 38 °C), uterine tenderness, and abnormal fetal heart rate were considered to exclude intra-amniotic infection (IAI). In addition, accompanying laboratory criteria such as elevated C-reactive protein levels and increased white blood cell counts were also considered. Microbiological criteria included the positive culture of pathogenic microorganisms in amniotic fluid. Patients meeting these criteria were excluded from our study. All patients admitted to our hospital with PPROM received standard antibiotic regimen within 6 h after admission. All PPROM patients received 1 g (g) oral azithromycin and 4*2 g intravenous ampicillin for 2 days. Antibiotic treatment was continued with amoxicillin 3*500 mg orally for 5 days. All patients received standard antenatal corticosteroid therapy consisting of two doses of 12 mg betamethasone administered intramuscularly every 24 h, in accordance with institutional protocol [12]. During corticosteroid therapy, all patients also received tocolytic therapy with oral nifedipine. The nifedipine protocol consisted of an initial dose of 20 mg followed by 10 mg every 6 h for 48 h. Blood samples for markers of inflammation, including CRP, were taken at admission and before initiation of corticosteroid and tocolytic therapy. Furthermore, magnesium sulfate treatment for fetal neuroprotection was administered between 24 + 0 and 29 + 6 weeks of gestation according to international guidelines [13].

### Data collection and laboratory procedures

Patient data were collected from medical records and the hospital’s information management system. Data on maternal age, gravidity, parity, pregestational BMI (kg/m^2^), presence/types of maternal chronic diseases, rates of in vitro fertilization (IVF) pregnancies, the history of previous preterm delivery, gestational age at diagnosis (week), maternal hemoglobin (Hb)(g/dl), leukocyte (10^9^/L), lymphocyte (10^9^/L), neutrophil (10^9^/L), platelet (10^9^/L), C-reactive protein (CRP) (mg/dL), time interval from diagnosis to delivery (days), gestational age at delivery (week), birth weight (g), 1^st^ and 5^th^ minute Apgar scores and route of delivery were obtained from patients’ medical files and compared between the subgroups. All blood parameters were evaluated after hospitalization before any treatment was started. The time interval between serial CRP values and CRP values requested at follow-up of patients was recorded. CRPv was calculated as the difference between CRP2 (mg/L) and CRP1 (mg/L) divided by the time (in hours) between the two results. CRP levels were measured at two consecutive time points to monitor changes in the inflammatory response over time. The first CRP sample was collected at the time of the patient’s presentation (0 h), and the second CRP sample was collected at least 18–24 h after presentation, taking into account the biological half-life of CRP (~ 19 h). IBI = CRP (mg/dL) × neutrophils (10^9^/L)/lymphocytes (10^9^/L), calculated using the complete blood cell count at the first hospitalization.

### Data management and analysis

In our study, demographic data were compared between the groups determined according to the timing of delivery, and the statistical differences between the groups were analyzed by comparing inflammatory indices. The relationship of these indices with perinatal and neonatal outcomes was comprehensively evaluated.

### Statistical analysis

Statistical analysis was conducted using IBM Corporation SPSS version 22.0 (IBM Corporation, Armonk, NY, USA). The Kolmogorov–Smirnov test was used to analyze the conformity to normal distribution. Descriptive statistics for continuous variables were presented as “mean ± standard deviation’ for those showing normal distribution and “median (interquartile range)” for those not showing normal distribution. Categorical variables were compared using the Chi-square test or Fisher’s exact test. Continuous variables were compared using the independent sample t-test and Mann–Whitney *U* test, depending on whether they showed a normal distribution. The receiver operating characteristic (ROC) curve was applied to determine the optimal cutoff values according to the Youden index by calculating and comparing the areas under the curve (AUC). In our study, multiple ROC analyses were performed, and statistical adjustments were made to prevent an increase in the type I error rate associated with multiple comparisons. *p* values were adjusted using the Benjamini–Hochberg (BH) method. A model was established with logistic regression analysis to predict birth within 48 h and 7 days, the results were reported with odds ratio (OR), 95% confidence interval (CI) and *p* value. Potential confounding variables such as BMI, parity, and cervical dilation were included in the model. Statistical significance for all tests was defined as a *p* value less than 0.05.

### Ethics and consents

The study was conducted in accordance with the principles stated in the Declaration of Helsinki and ethical approval was obtained from Ankara Etlik City Hospital Ethics Committee (approval number: AESH-BADEK-2025-0087). Considering the retrospective nature of the study, Ankara Etlik City Hospital Ethics Committee granted an exemption for informed consent.

## Results

In this study, maternal characteristics of the patients hospitalized due to PPROM who delivered before 48 h (*n* = 167, 49.5%) and those who delivered after 48 h (*n* = 170, 50.5%) are compared in Table 1. Maternal age, gravida, parity, BMI, and IVF pregnancy rates were similar between the groups (*p* = 0.182, *p* = 0.105, *p* = 0.108, *p* = 0,053, *p* = 0.408, respectively). However, gestational week at hospitalization, hemoglobin levels, leukocyte and neutrophil counts, and fibrinogen levels were significantly different (*p* =  < 0.001, *p* = 0.002, *p* = 0.013, *p* = 0.004 and *p* =  < 0.001, respectively). Lymphocyte count, platelet count and albumin levels were similar between the groups (*p* = 0.849, *p* = 0.747, *p* = 0.586, respectively). A significant difference was also found between the groups in terms of cervical dilatation; the mean cervical dilatation was 2 (2) cm in the group delivering before 48 h, whereas this value was 1 (1) cm in the group delivering after 48 h (*p* < 0.001).

**Table 1:** Characteristics of patients hospitalized due to threatened preterm premature rupture of membrane delivery who gave birth before and after 48 h

	Delivery before 48 h *n* = 167 (49.5%)	Delivery after 48 h *n* = 170 (50.5%)	*p* value
Maternal age (year)	28 ± 6	29 ± 6	0.182^a^
Gravida	2 (2)	2 (3)	0.105^b^
Parity	1 (2)	1 (2)	0.108^b^
In vitro fertilization	9 (%5.4)	6 (3.5%)	0.408^c^
BMI (kg/m^2)^	26.9 ( 5.8)	28.7 (6.8)	0.053^b^
Gestational week at hospitalization	33 (3)	30 (5)	**< 0.001**^b^
Hemoglobin (g/dl)	11.8 (1.6)	11.4 (2.0)	**0.002**^b^
White blood cell count (10^9^/L)	12.8 (5.9)	12.2 (4.6)	**0.013**^b^
Lymphocyte count (10^9^/L)	1.9 (1.4)	1.8 (0.9)	0.849^b^
Neutrophil count (10^9^/L)	10.1 (6.3)	8.9 (4.5)	**0.004**^b^
Platelet count (10^9^/L)	257 (99)	254 (74)	0.747^b^
Albumin (g/dl)	36.8 (3.6)	37.1 (2.8)	0.586^b^
Fibrinogen (mg/dL)	527 (135)	482 (112)	**< 0.001**^b^
Cervical dilatation	2 (2)	1 (1)	**< 0.001**^b^

Data are expressed as mean ± SD or median (interquartile range) where appropriate. A *p* value of < 0.05 indicates a significant difference and statistically significant *p* values are in bold

*BMI* body mass index, *SD* standard deviation

^a^Student *T*-test

^b^Mann–Whitney *U* test

^c^Pearson Chi-square

Neonatal characteristics and results are presented in Table 2. Gestational week at delivery, cesarean section, the mean birth weight was rate was statistically significant between the groups (*p* = 0.003, *p* = 0.004 and *p* = 0.002 respectively). No significant difference was observed between the groups in terms of Apgar scores at 1 min (*p* = 0.075) and 5 min (*p* = 0.069). The rate of neonatal intensive care unit (NICU) hospitalization, transient neonatal tachypnea (TTN) and continuous positive airway pressure (CPAP) application was different between the groups, and this difference was statistically significant (*p* = 0.022, *p* = 0.002 and *p* = 0.019, respectively). There was no significant difference in the rates of neonatal sepsis (*p* = 0.687), fetal distress (*p* = 0.496) and RDS (*p* = 0.353) between the groups. No significant difference was observed between the groups in terms of mechanical ventilation requirement (*p* = 0.651), phototherapy rate (*p* = 0.711) and neonatal hypoglycaemia (*p* = 0.388). There was no difference in the rate of IVH and NEC between the groups (*p* = 0.061, *p* = 0.123).

**Table 2:** Birth characteristics and neonatal outcomes of the newborns

	Delivery before 48 h *n* = 167 (49.5%)	Delivery after 48 h *n* = 170 (50.5%)	*p* value
Gestational age at delivery (week)	33 (3)	32 (2.1)	**0.003**^b^
Cesarean section	84 (50.4%)	112 (65.9%)	**0.004**^c^
Birth weight (gram)	1917 ± 592	1715 ± 605	**0.002**^a^
Apgar score at 1^st^ minute	7 (2)	7 (3)	0.075^b^
Apgar score at 5^th^ minute	9 (2)	8 (2)	0.069^b^
NICU admission	120 (71.9%)	140 (82.4%)	**0.022**^c^
Umbilical cord pH	7.30 (0.14)	7.30 (0.14)	0.739^b^
Transient tachypnea of the newborn	36 (21.6%)	63 (37.1%)	**0.002**^c^
Neonatal sepsis	20 (12.0%)	18 (10.6%)	0.687^c^
Fetal distress	27 (16.2%)	23 (13.5%)	0.496^c^
Respiratory distress syndrome	58 (34.7%)	51 (30.0%)	0.353^c^
Continues positive airway pressure	72 (43.1%)	95 (55.9%)	**0.019**^c^
Mechanical ventilation	56 (33.5%)	61 (35.9%)	0.651^c^
Phototherapy for neonates	26 (15.6%)	29 (17.1%)	0.711^c^
Neonatal hypoglycemia	15 (9.0%)	11 (6.5%)	0.388^c^
Interventricular hemorrhage	0 (0%)	5 (2.9%)	0.061^c^
Necrotizing enterocolitis	0 (0%)	4 (2.4%)	0.123^c^

Data are expressed as mean ± SD or median (interquartile range) where appropriate. A *p* value of < 0.05 indicates a significant difference and statistically significant p values are in bold

*NICU* neonatal intensive care unit

^a^Student *T*-test

^b^Mann–Whitney *U* test

^c^Pearson Chi-square test

Table 3 shows the comparison of laboratory and index results of the groups. Accordingly, the first CRP value and second CRP value were different between the groups (*p* = 0.001, *p* = 0.001). CRP velocity was 0.33 (0.9) in the group who delivered before 48 h and 0.10 (0.4) in the group who delivered after 48 h, and this difference was significant (*p* < 0.001). IBI value was 44.2 (135.6) in the group who gave birth before 48 h and 37.5 (57.7) in the group who gave birth after 48 h, and this difference was statistically significant (*p* = 0.047). The first CRP value was 9.1 (14.2) mg/L in the group who delivered before 7 days and 6.9 (8.9) mg/L in the group who delivered after 7 days, and this difference was statistically significant (*p* = 0.045). The second CRP value was 13.3 (32.4) mg/L in the group who gave birth before 7 days and 14.5 (29.6) mg/L in the group who gave birth after 7 days, and this difference was not significant (*p* = 0.730). CRP velocity was 0.16 (0.66) in the group who gave birth before 7 days and 0.14 (0.50) in the group who gave birth after 7 days, and this difference was not significant (*p* = 0.630). Inflammatory marker index (IBI) was 44.0 (127.1) in the group giving birth before 7 days and 34.5 (45.6) in the group giving birth after 7 days, and this difference was statistically significant (*p* = 0.024).

**Table 3:** Comparison of laboratory and index results of the groups

	Delivery before 48 h *n* = 167 (49.5%)	Delivery after 48 h *n* = 170 (50.5%)	*p* value	Delivery before 7 days *n* = 250 (74.2%)	Delivery after 7 days *n* = 87 (25.8%)	*p* value
CRP first (mg/L)	9.1 (12.9)	7.5 (11.2)	**0.027**^a^	9.1 (14.2)	6.9 (8.9)	**0.045**^a^
CRP second (mg/L)	19.3 (39.3)	10.1 (26.7)	**0.001**^a^	13.3 (32.4)	14.5 (29.6)	0.730^a^
Velocity of CRP	0.33 (0.9)	0.10 (0.4)	**< 0.001**^a^	0.16 (0.66)	0.14 (0.50)	0.630^a^
IBI	44.2 (135.6)	37.5 (57.7)	**0.047**^a^	44.0 (127.1)	34.5 (45.6)	**0.024**^a^

Data are expressed as median (interquartile range). A *p* value of < 0.05 indicates a significant difference and statistically significant *p* values are in bold

*CRP* C-reactive protein, *IBI* inflammatory burden index

^a^Mann–Whitney *U* test

**Fig. 1:**
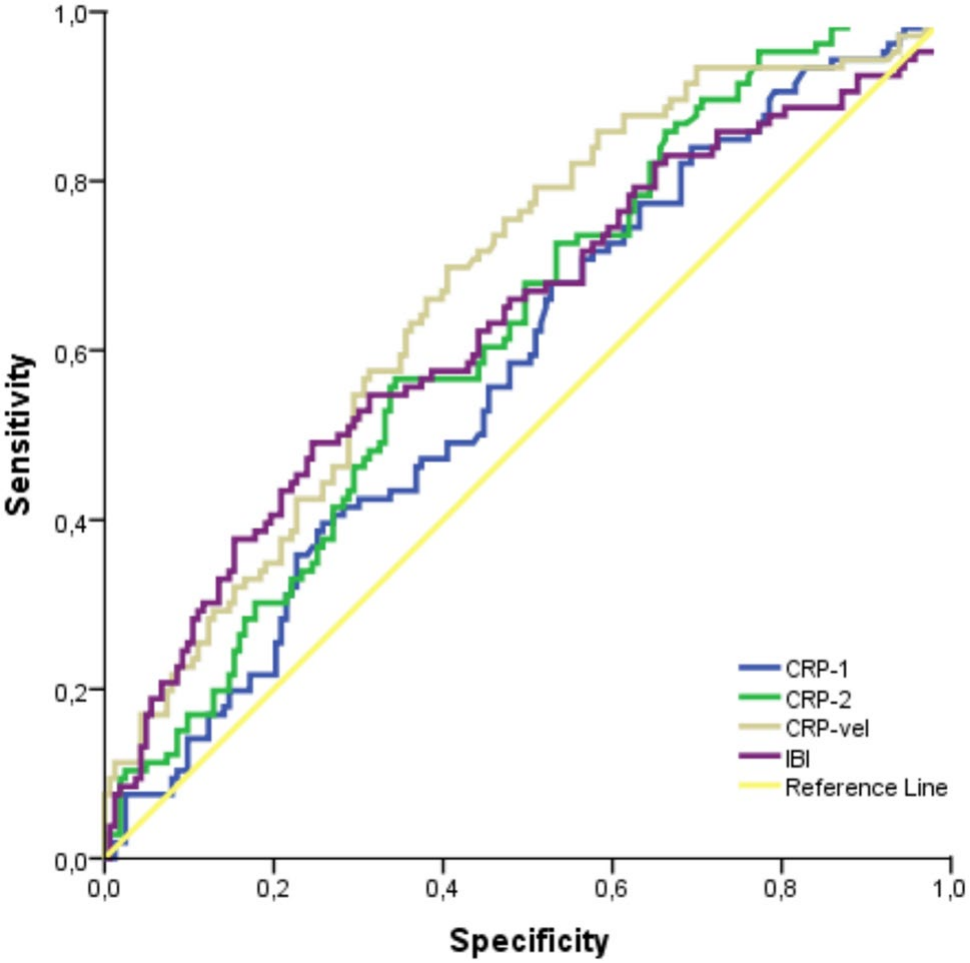
ROC curve of CRP (first), CRP (second), velocity of CRP and IBI in patients with preterm premature rupture of membranes for prediction of delivery in 48 h

**Fig. 2:**
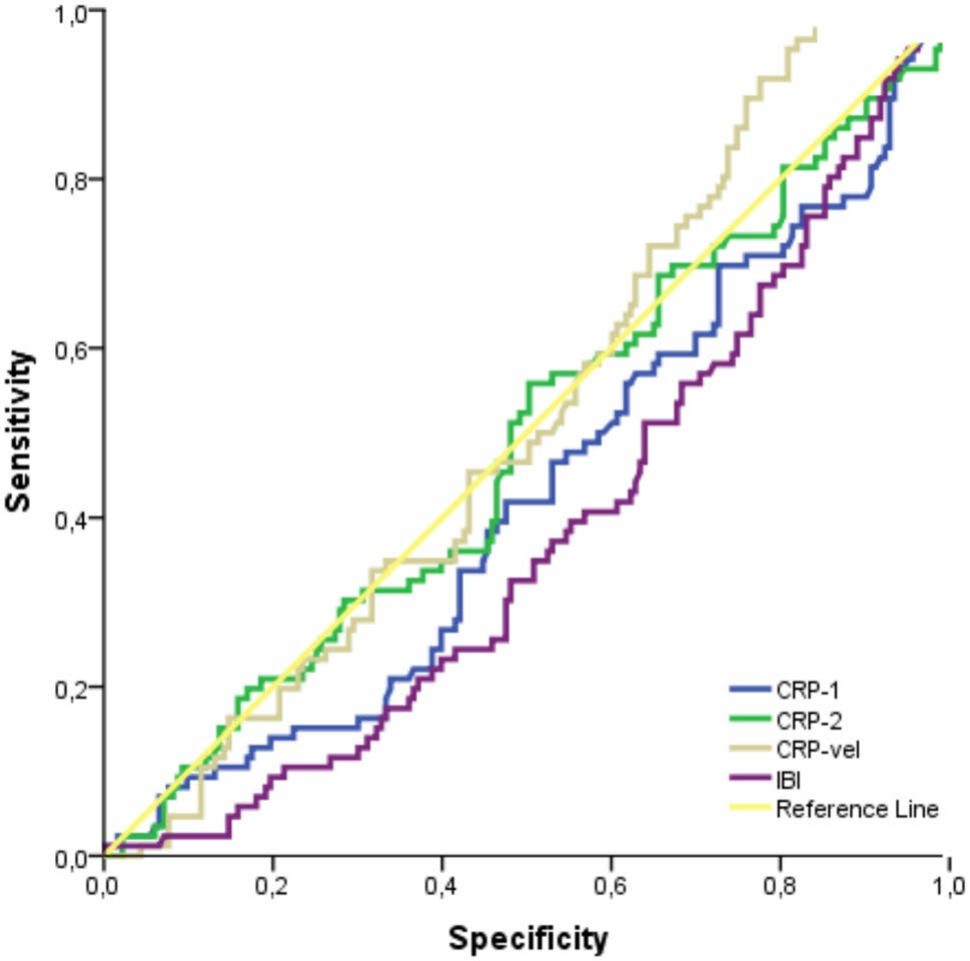
ROC curve of CRP (first), CRP (second), velocity of CRP and IBI in patients with preterm premature rupture of membranes for prediction of delivery in 7 days

Table 4 and Fig. 1 present the evaluation of CRP (first), CRP (second), CRP velocity and IBI by ROC analysis to predict delivery within 48 h in PPROM patients at risk of preterm delivery. With a first CRP measurement > 8.61, the positive predictive value (PPV) was 0.43, the negative predictive value (NPV) was 0.65, the sensitivity was 55% and the specificity was 53%, with an AUC of 0.580 and a 95% confidence interval of 0.51–0.65 (*p* = 0.027). For the second CRP measurement with a cutoff value > 10.08, PPV was 0.47, NPV 0.70, sensitivity 68%, specificity 50%, AUC 0.623, 95% confidence interval 0.56–0.69 and p value 0.001. With a cutoff value of CRP velocity > 0.17, PPV was 0.46, NPV 0.85, sensitivity 70%, specificity 67%, AUC 0.675, 95% confidence interval 0.61–0.74, and *p* value < 0.001. Finally, with a cutoff value of IBI > 35.8, PPV was 0.53, NPV 0.55, sensitivity 61%, specificity 50%, AUC 0.563, 95% confidence interval 0.50–0.62 and p value 0.047. According to these findings, especially the second CRP measurement and CRP velocity were found to be more accurate in predicting delivery within 48 h. In this study, two separate multivariate logistic regression analyses were performed to evaluate markers potentially associated with birth within 48 h and 7 days. The results of these analyses are presented in Tables 5 and 6. According to the analysis aimed at predicting birth within 48 h, CRP level was found to be an important predictive factor (OR: 0.633, 95% CI 0.495–0.811, *p* < 0.001). In contrast, the first and second CRP measurements and IBI were not found to be statistically significant in predicting birth within 48 h (*p* = 0.586, *p* = 0.099, and *p* = 0.074, respectively). In the analysis aimed at predicting birth within 7 days, no variable showed a statistically significant effect. Table 7 and Fig. 2 show the evaluation of CRP (first), CRP (second), CRP velocity and IBI by ROC analysis to predict delivery within 7 days in PPROM patients at risk of preterm labor. For the first CRP value with a cutoff value > 7.56, the positive predictive value (PPV) was 0.71, the negative predictive value (NPV) was 0.35, sensitivity 58%, specificity 50%, AUC 0.57, 95% confidence interval 0.50–0.65 and *p* value 0.045. The results of the analyses for the second CRP value and CRP velocity were not given, and the *p* values for these variables were 0.730 and 0.630, respectively. For the IBI value, the PPV was 0.79, NPV 0.32, sensitivity 67%, specificity 50%, AUC 0.62, 95% confidence interval 0.55–0.69 and *p* value 0.002 with a cutoff value > 37.3. According to these findings, first CRP and IBI gave significant results in terms of predicting labor within 7 days, while no significant prediction was provided for second CRP and CRP velocity.

**Table 4:** Evaluation of CRP (first), CRP (second), velocity of CRP and IBI in patients with preterm premature rupture of membranes for prediction of delivery in 48 h using ROC analysis

	PPV	NPV	Cutoff*	Sensitivity	Specificity	AUC	%95 CI	*p* value
CRP (first)	0.43	0.65	> 8.61	55%	53%	0.580	0.51–0.65	**0.027**
CRP (second)	0.47	0.70	> 10.08	68%	50%	0.623	0.56–0.69	**0.001**
Velocity of CRP	0.46	0.85	> 0.17	70%	67%	0.675	0.61–0.74	**< 0.001**
IBI	0.53	0.55	> 35.8	61%	50%	0.563	0.50–0.62	**0.047**

A *p* value of 0.05 indicates a significant difference and statistically significant *p* values are in bold

^*^Cutoff values were found according to Youden index

*PPV* positive predictive value, *NPV* negative predictive value, *AUC* area under the curve, *CI* confidence interval, *CRP* C-reactive protein, *IBI* inflammatory burden index

**Table 5:** Regression analysis to predict the delivery in 48 h

Variable	OR (95% CI)	*p* value
CRP (first)	0.996 (0.983–1.010)	0.586
CRP (second)	0.995 (0.989–1.001)	0.099
Velocity of CRP	0.633 (0.495–0.811)	**< 0.001**
IBI	0.999 (0.998–1.000)	0.074

A *p* value of 0.05 indicates a significant difference and statistically significant *p* values are in bold

*CI* confidence interval, *CRP* C-reactive protein, *IBI* inflammatory burden index

**Table 6:** Regression analysis to predict the delivery in 7 days

Variable	OR (95% CI)	*p* value
CRP (first)	1.006 (0.991–1.022)	0.404
IBI	1.001 (0.999–1.002)	0.290

*CI* confidence interval, *CRP* C-reactive protein, *IBI* inflammatory burden index

**Table 7:** Evaluation of CRP (first), CRP (second), velocity of CRP and IBI in patients with preterm premature rupture of membranes for prediction of delivery in 7 days using ROC analysis

	PPV	NPV	Cutoff*	Sensitivity	Specificity	AUC	%95 CI	*p* value
CRP (first)	0.71	0.35	7.56	58%	50%	0.57	0.50–0.65	**0.045**
CRP (second)	–	–	–	–	–	–	–	0.730
Velocity of CRP	–	–	–	–	–	–	–	0.630
IBI	0.79	0.32	37.3	67%	50%	0.62	0.55–0.69	**0.002**

A *p* value of 0.05 indicates a significant difference and statistically significant *p* values are in bold

^*^Cutoff values were found according to Youden index

*PPV* positive predictive value, *NPV* negative predictive value, *AUC* area under the curve, *CI* confidence interval, *CRP* C-reactive protein, *IBI* inflammatory burden index

## Discussion

In this study, we evaluated the role of CRPv and IBI, which are candidates to be a new marker in predicting labor latent period and neonatal outcomes in PPROM cases. Although these two markers have been evaluated in different clinical scenarios in previous studies, to our knowledge, no study has been conducted in obstetrics. In line with the findings of our study, we found that CRPv and IBI values were significantly higher in the group of patients with a latent period shorter than 48 h, suggesting that serial CRP evaluation and inflammatory index including CRP, neutrophils and lymphocytes are promising in predicting the time of delivery in patients presenting with PPROM. Similarly, we found that the CRP value and IBI values at presentation were significantly higher in the patient group with a latent period of less than 7 days. Furthermore, unlike other studies in the literature, we determined cutoff values for predicting the time of delivery by assessing the rate of CRP change in PPROM cases. We demonstrated that this cutoff value could help in appropriately selecting patients for early treatment and intervention.

Amniotic inflammation, the underlying mechanism of PPROM, can be diagnosed using interleukin-6 (IL-6), which is considered one of the strongest and best-characterized biomarkers in the literature. Romero et al. have demonstrated in numerous studies that IL-6 has high sensitivity and specificity in predicting both microbial invasion and adverse perinatal outcomes [14, 15]. However, the IL-6 test is not readily available for routine clinical use in many developing countries. Based on this reality, our study evaluated CRP velocity, a systemic inflammation marker that is more easily accessible and less expensive in clinical practice. Results of our study suggest that parameters such as CRP velocity could serve as a practical alternative for predicting intra-amniotic inflammation, particularly in healthcare systems where access to advanced biomarkers like IL-6 is limited. Although vaginal secretions and amniotic fluid samples were not used in our study, inflammatory markers obtained from these materials, particularly IL-6 and MMP-8, are known to have significant prognostic value in the early diagnosis of obstetric complications such as intra-amniotic inflammation and PPROM. In the studies by Romero et al., the non-invasive nature of these markers was emphasized, and it was demonstrated that they can be effectively integrated into clinical practice, enabling more sensitive diagnosis in the early stages of inflammation [16]. These parameters obtained from vaginal secretions offer important advantages in identifying conditions requiring early intervention and enable the avoidance of unnecessary invasive procedures.

Previous studies have examined the relationship between neutrophil, lymphocyte and CRP levels and intra-amniotic inflammation (IAI). In a study by Musilová et al. [5], maternal serum CRP concentrations were higher in women with MIAC and IAI compared to those without. Prediction of the time of birth is very important for the prevention of many complications related to prematurity [17]. Therefore, preventing neonatal complications should be our primary goal. Furthermore, prediction of neonatal outcomes is critical for the provision of appropriate NICU facilities for neonates. Ryu et al. [18] found that CRP levels were significantly higher in the PPROM patient group with a latent period shorter than 72 h. While these studies mostly evaluate inflammatory biomarkers based on the value of a single time point, in our study, we used the rate of change in CRP levels over time and the IBI. CRPv is a promising biomarker that may enhance the diagnostic and prognostic capabilities of traditional CRP measurements. CRPv has previously been evaluated in studies to distinguish between bacterial and viral infection, predict appendicitis and determine cardiovascular risk [19–21]. Largman-Chalamish et al. [21] have shown that the CRPv value during an acute febrile illness can be used to quickly differentiate between bacterial and viral infection. This feature could potentially accelerate the provision of appropriate therapeutic management. When these previous studies were analyzed, it was understood that CRPv could be used not only in infective conditions but also in the prediction of prognosis due to underlying inflammatory diseases. Considering these considerations, we decided to use CRPv in our study to evaluate the outcomes of the inflammatory process in patients presenting with PPROM with or without infection. In our retrospective study, we divided PPROM patients into two groups according to their latent period. We found that neutrophil count, CRPv and IBI values were significantly higher in PPROM patients with a latent period of less than 48 h compared to the patient group with a latent period of more than 48 h. Patients with CRPv levels above 0.17 were more likely to give birth in less than 48 h. The findings suggest that CRPv can serve as a reliable threshold for accurate estimation of time of labor (AUC 0.675, 95% CI 0.61–0.74, *p* =  < 0.001). The test showed 70% sensitivity and 67% specificity at the cutoff value. Our results indicate that CRPv, as a dynamic indicator of inflammatory progression, offers more predictive accuracy than a solitary CRP measurement. This corresponds with research indicating that serial inflammatory markers may more accurately represent the development of infection than static levels.

IBI is an index developed to predict prognosis and manage treatment in various diseases. This index is defined as a combination of inflammatory markers such as C-reactive protein and neutrophil/lymphocyte ratio and is considered a potential tool for predicting clinical outcomes in a wide range of conditions from cancer to cardiovascular disease. Its ability to reflect systemic inflammation makes it a valuable tool for clinicians in assessing patient outcomes and determining treatment strategies. This index has been mostly studied in cancer patients in the literature, and no study on pregnant women has been found [22, 23]. In our study, IBI values were significantly higher in the group that gave birth before 48 h compared to the group that gave birth after 48 h (*p* = 0.047). ROC analysis showed 61% sensitivity, 50% specificity, and an AUC value of 0.563. These results indicate that IBI is not sufficient on its own for an accurate clinical diagnosis, but it may be clinically significant. The AUC value of IBI at this level suggests that the biomarker may support clinical decisions with limited accuracy but does not provide a reliable diagnosis when used alone. While these findings do not rule out the potential clinical relevance of IBI, they emphasize the importance of using it in combination with other biochemical markers to achieve better accuracy. Considering the multifactorial nature of PPROM and the role of membrane rupture in its pathophysiology, the inclusion of IBI in combination with other biomarkers in multi-biomarker panels or risk models could enhance predictive accuracy and contribute to clinical decision-making processes. In this context, further large-scale prospective studies are needed to validate the role of IBI.

In our study, we found that CRPv and IBI played a significant role in predicting the time of labor. These findings suggest that biomarkers can be used as a potential tool in obstetric management. If these biomarkers reach high levels in the early term, earlier initiation of corticosteroid therapy may reduce the complications of premature births and support the maturation process of the fetus.

As these biomarkers can predict the risk of preterm birth, they may be important in personalizing treatment strategies. In other words, these biomarkers not only predict the timing of labor, but may also help to shape treatment decisions so that the pregnant woman and fetus can achieve the best clinical outcomes.

The strengths of our study include being conducted in a single center, having a standard follow-up protocol, evaluating many parameters together and selecting a homogeneous PPROM group. The retrospective nature of this study brings some limitations. This may affect the reliability and generalisability of the results. Second, this study is the absence of amniotic fluid analysis for direct inflammatory marker evaluation. Amniocentesis was not performed due to ethical and institutional guidelines, which discourage its use in PPROM cases unless clinically indicated. Since the data used in the study belong to a specific time period and institution, the applicability of the findings to other populations or different clinical settings may be limited. Despite these limitations, the findings of this study provide important information for the management of PPROM and prediction of labor timing. However, future prospective, randomized controlled trials and larger sample sizes are needed to validate these findings and integrate them more effectively into clinical practice.

## Conclusion

This study demonstrated that novel systemic inflammatory biomarkers such as CRPv and IBI may play an important role in predicting the latent period until delivery in pregnancies with PPROM. Our findings suggest that these biomarkers are effective in assessing the maternal and fetal inflammatory response and may contribute to clinical decision-making in the management of pregnancy after PPROM. In conclusion, CRPv and IBI may offer an innovative approach to the management of PPROM, contributing to improved maternal and fetal outcomes.

## Data Availability

No datasets were generated or analysed during the current study.
